# Supplementary material for: Discovering human diabetes-risk gene function with genetics and physiological assays
Source: Nat Commun. 2018 Sep 21;9:3855. doi: 10.1038/s41467-018-06249-3 (PMC6155000; doi:10.1038/s41467-018-06249-3)
Supplement: Supplementary file 1 — Supplmentary Information [file 41467_2018_6249_MOESM1_ESM.pdf]

Supplementary Information

Discovering human diabetes-risk gene function with genetics and physiological assays.

Heshan Peiris, Sangbin Park, Shreya Louis, Xueying Gu, Jonathan Y. Lam, Olof Asplund, Gregory C. Ippolito, Rita Bottino, Leif Groop, Haley Tucker, Seung K. Kim.

Supplementary Figure 1 (related to Figure 1)

a

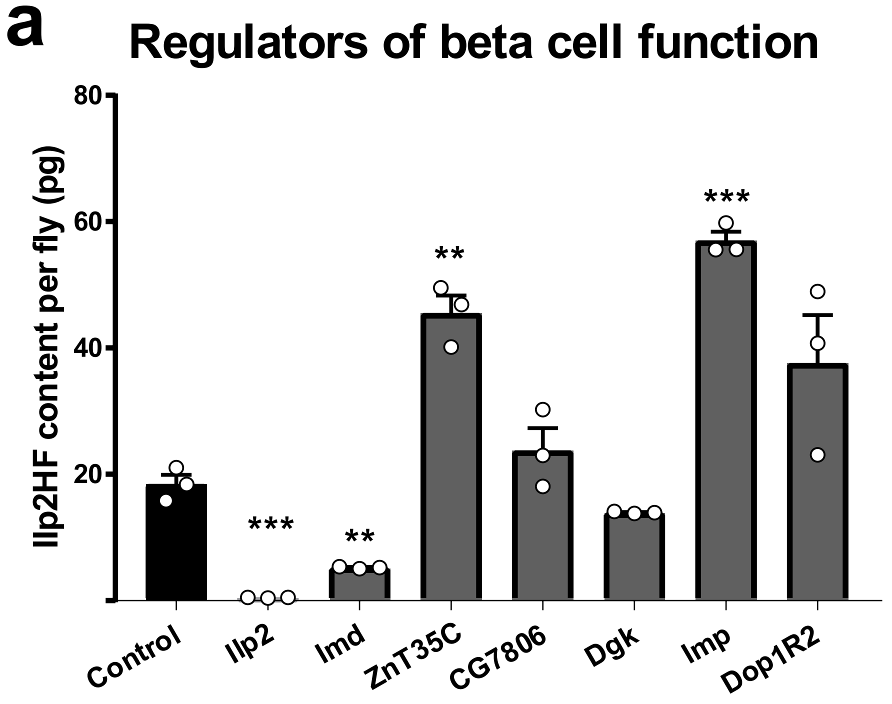

c

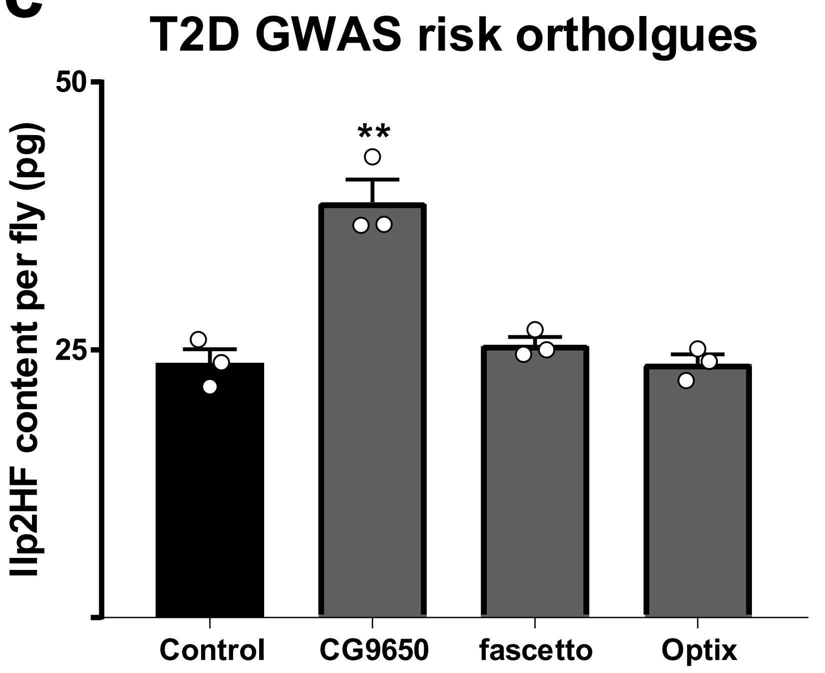

b

T2D GWAS candidate gene orthologs screened

Drosophila Gene	Human Orthologue
Hnf4	HNFA
foxo	FOXO1
Wnk	HNF1B
tap	NEUROD1
Ork1	KCNK16
Rab3-GEF	MADD
groucho	TLE4
Vps13	VPS13C
Akt1	AKT2
KCNQ	KCNQ1
CG30340	MTNR1B
cry	CRY2
fkh	FOXA2
shep	RBMS1
Rpn7	PSMD6
CG43373	ADCY5
ST6Gal	ST6GAL1
wfs1	WFS1
CD12054	JAZF1
DOR	TP53INP1
Lar	PTPRD
tonalli	ZMIZ1
CG1812	KLHDC5
sprouty	SPRY
Dip-C	PEPD
MKp3	DUSP9

d

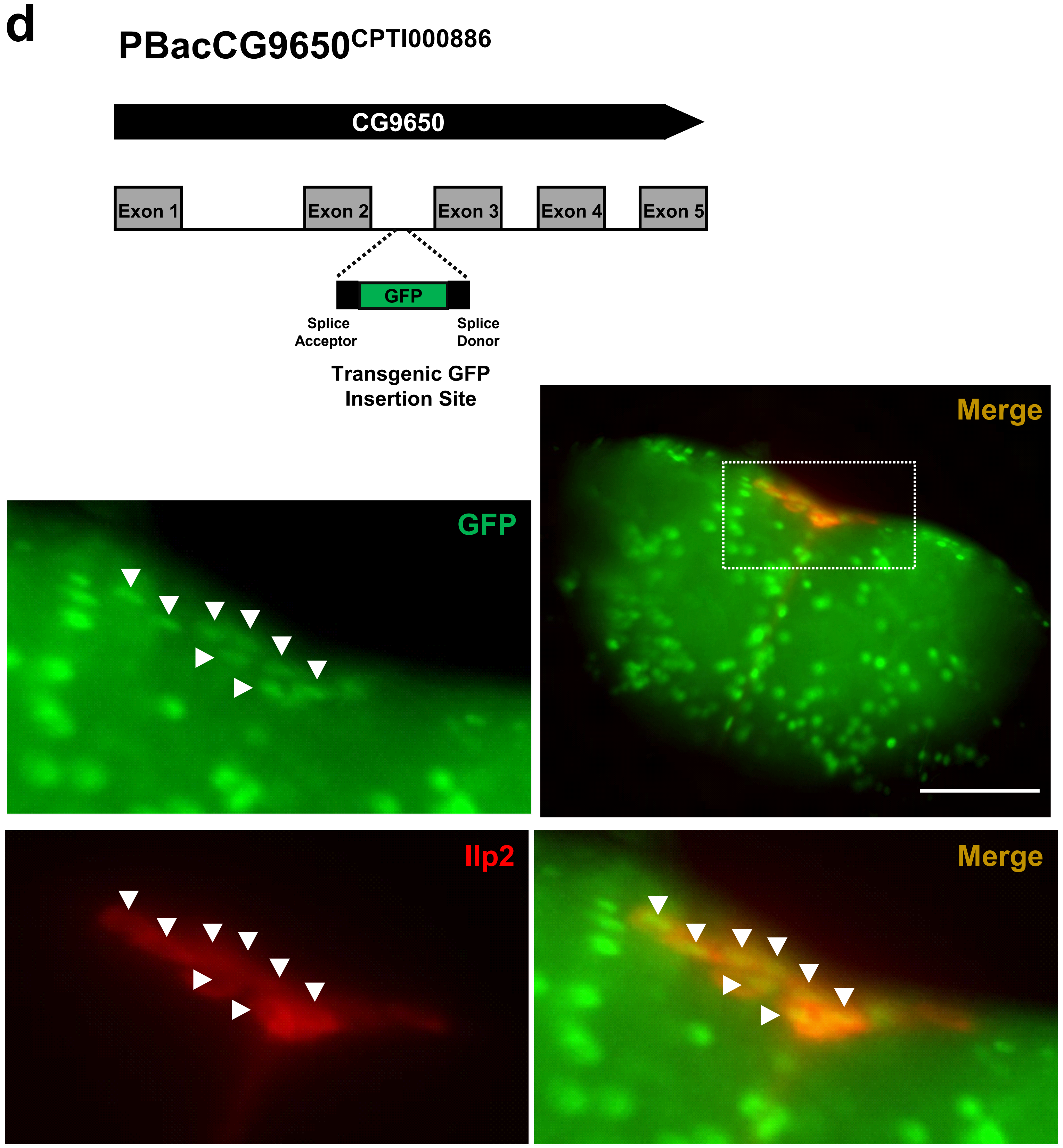

Supplementary Figure 1: **(a)** Total Ilp2HF content (picogram, pg) in ad libitum fed adult flies with IPC-specific RNAi knockdown of beta cell genes (n=3 independent experiments, sampled in duplicate).

(b) T2D GWAS candidate gene orthologs screened, 26 shown here. See figure 1c for prioritized list of 14 genes.

(c) Total Ilp2HF content (pg) in ad libitum fed adult flies with IPC-specific RNAi knockdown of CG9650, fascetto and Optix (n=3 independent experiments, sampled in duplicate).

(d) Schematic diagram of the PBacCG9650 CPTI000886 fly, showing the GFP insertion site within the CG9650 locus, immunostaining of adult Drosophila brains with antibodies recognizing GFP (green) or Ilp2 (red), scale bar = 100 μm.

Data presented as mean, error bars represent the standard error, and two-tailed t-tests were used to generate p values. ** $p<0.01$, *** $p<0.001$.

Supplementary Figure 2 (related to Figure 2)

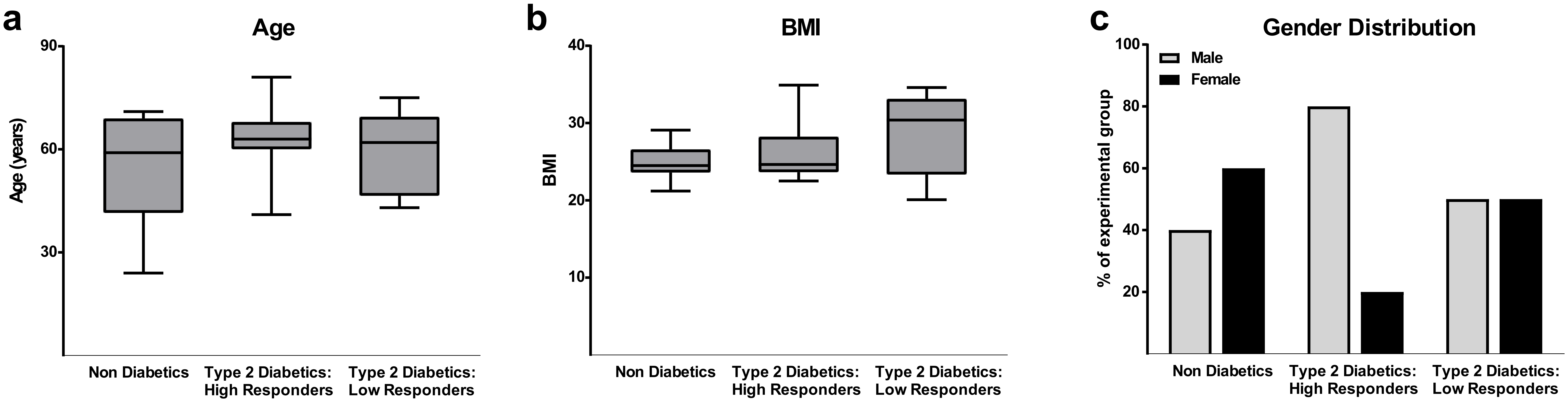

Supplementary Figure 2: **(a)** Mean donor age across the three defined groups.
(b) Mean body mass index (BMI) across the three defined groups.
(c) Gender distribution across the three defined groups.
Data obtained from a total of 30 donors (10 per group), presented as mean, error bars represent the minimum and maximum points, and two-tailed t-tests were used to generate p values.

Supplementary Figure 3 (related to Figure 3)

a

Unos ID	Age	Sex	BMI
AEBR210A	59	Male	27.2
AEBZ012	60	Male	37.9
AECP287	44	Male	31.2
AEDG094	49	Male	25.5
AEDT128A	26	Female	38.9

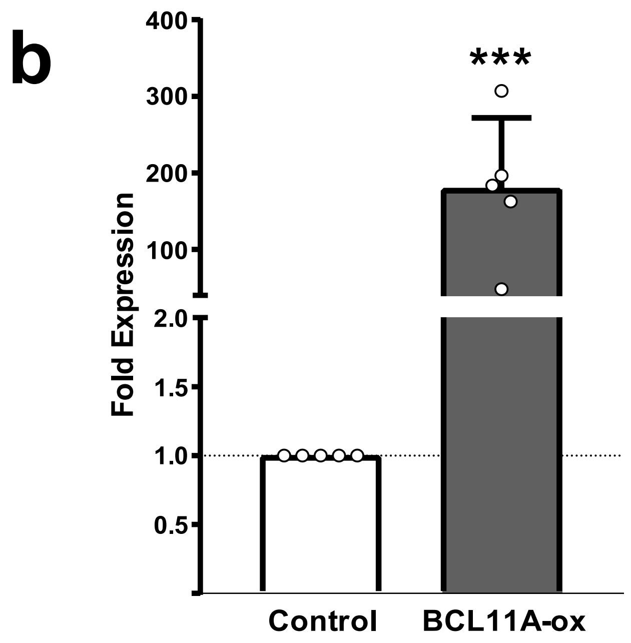

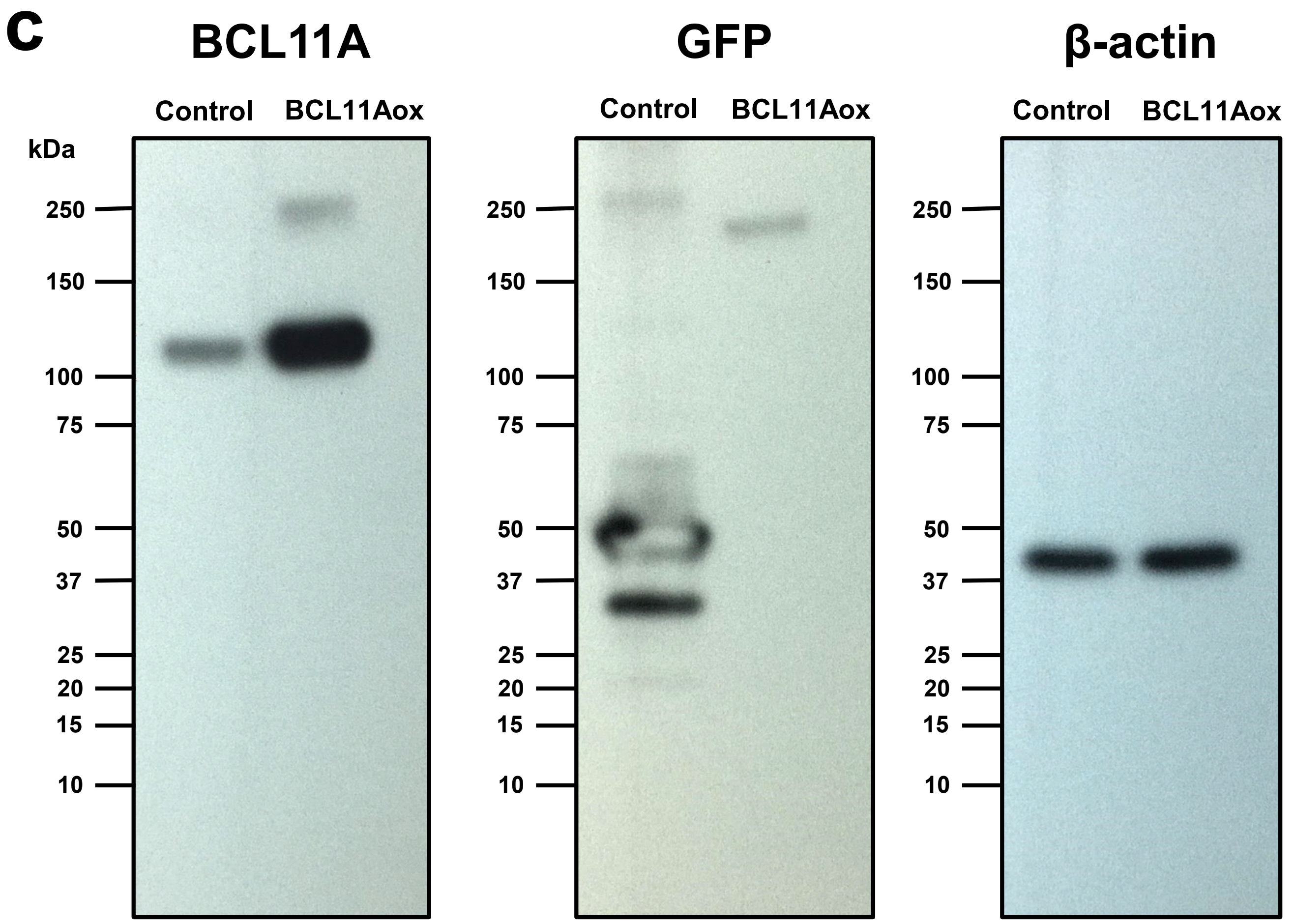

Supplementary Figure 3: **(a)** Donor information of the islets used to generate data presented in Figure 3. **(b)** BCL11A mRNA expression in human pseudo islets following transduction with control (white bar) or BCL11ox (grey bar) lentiviral vectors (n=5), data normalized to human beta actin. **(c)** Original scan of blots against BCL11A, GFP and β-actin shown in Figure 3d. Data presented as mean, error bars represent the standard error, and two-tailed t-tests were used to generate p values. *** p<0.001.

Supplementary Figure 4 (related to Figure 4)

a

Donor ID	Age	Sex	BMI
R204	62	Male	23.8
rHIP-112	41	Male	29.2
AEAN295	47	Female	31.1
AEBM372	65	Female	35.1

b

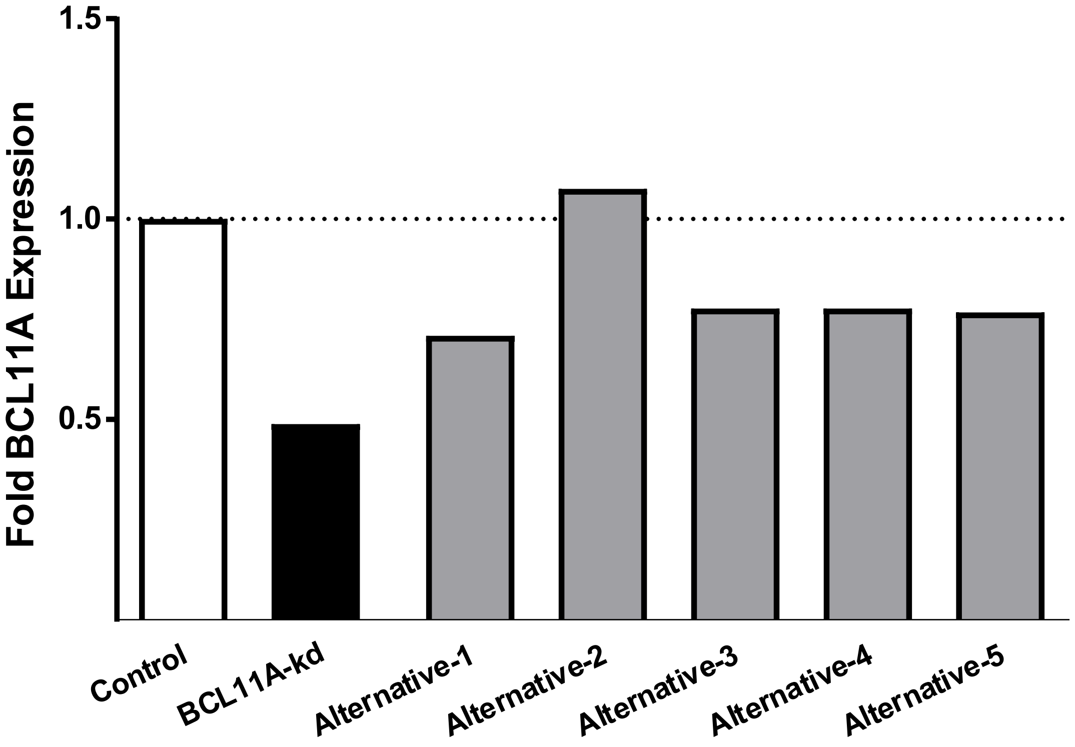

c

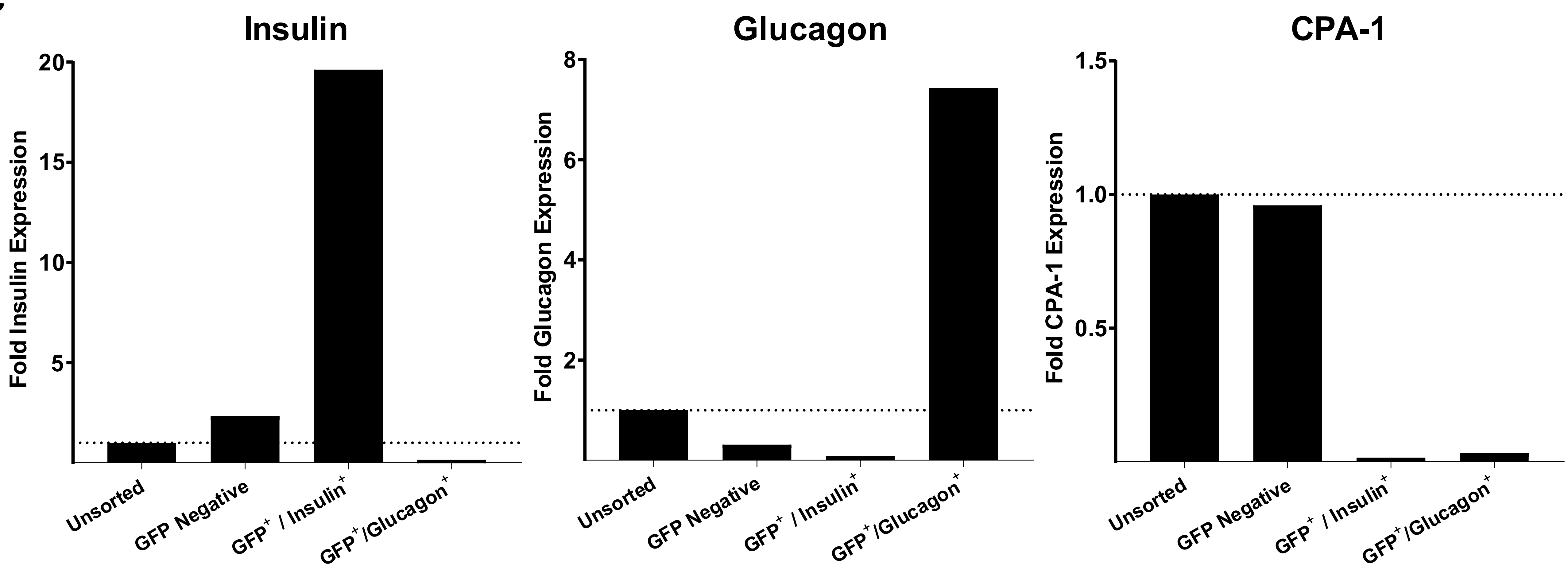

Supplementary Figure 4: **(a)** Donor information of the islets used to generate data presented in Figure 4.
(b) BCL11A mRNA expression in human pseudo islets following transduction with control (white bar), BCL11-kd (black bar) or alternative lentiviral vectors (grey bars) targeting different regions of the BCL11A gene (n=1), data normalized to human beta actin.
(c) Insulin, Glucagon or CPA-1 mRNA expression in unsorted, GFP negative, GFP+ Insulin+, GFP+ Glucagon+ human pseudo islet cells (n=1), data normalized to human beta actin.

Supplementary Figure 5 (related to Figure 5)

a

Donor ID	Age	Sex	BMI
ADE1444	33	Female	34.2
ADFN400	30	Female	35.8
ADF2083	24	Male	34.8
ADGZ426	45	Male	30.5

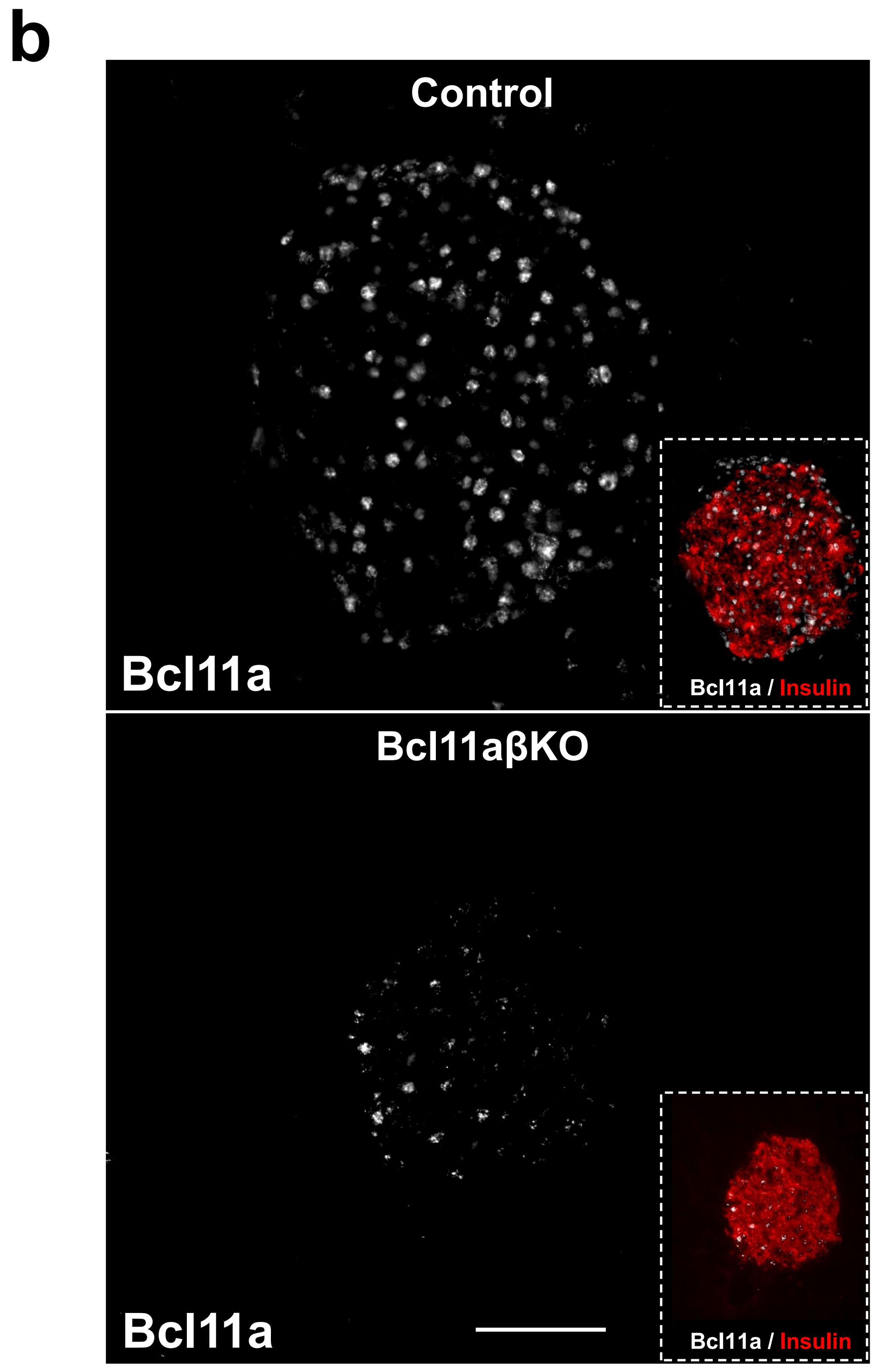

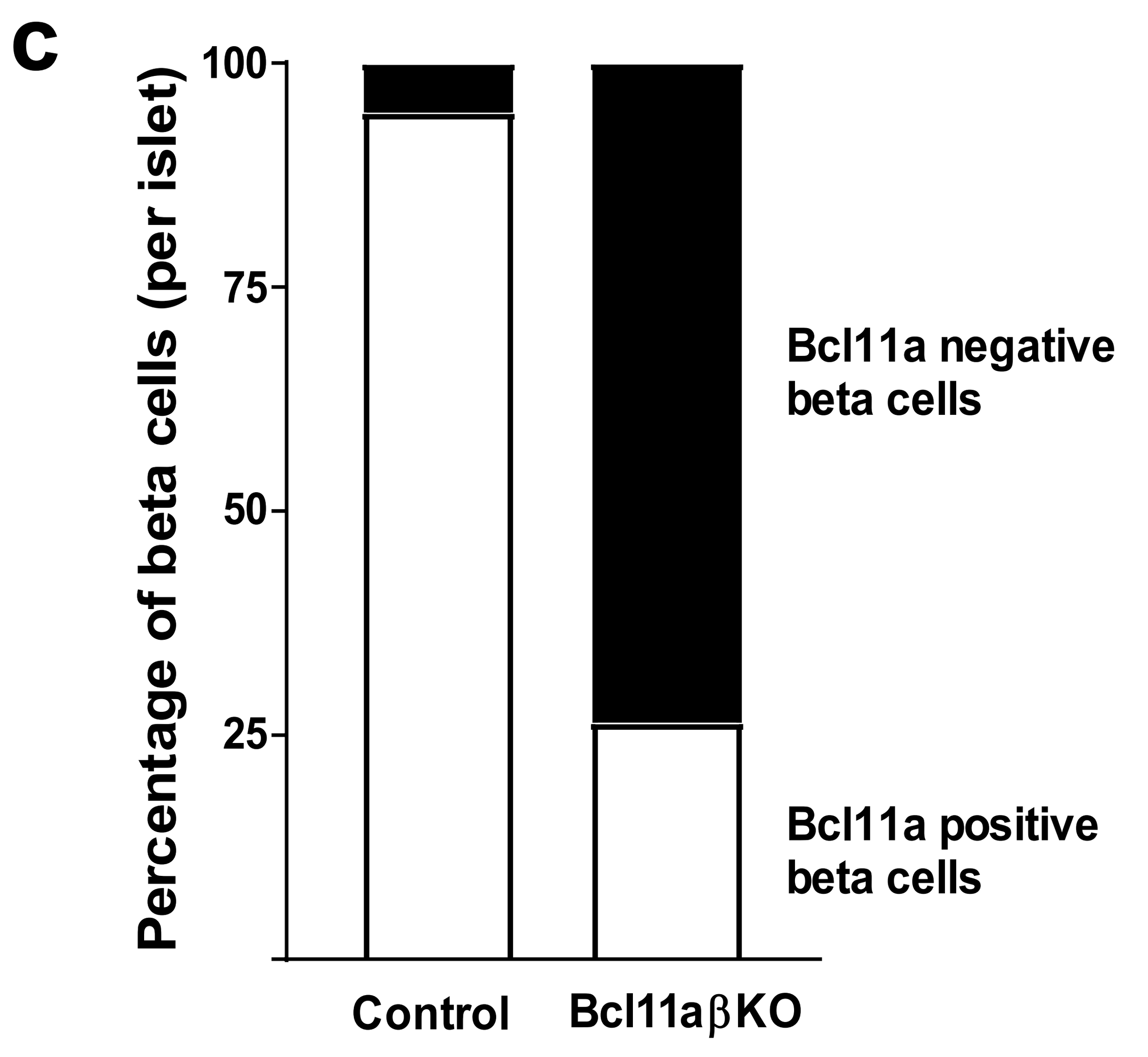

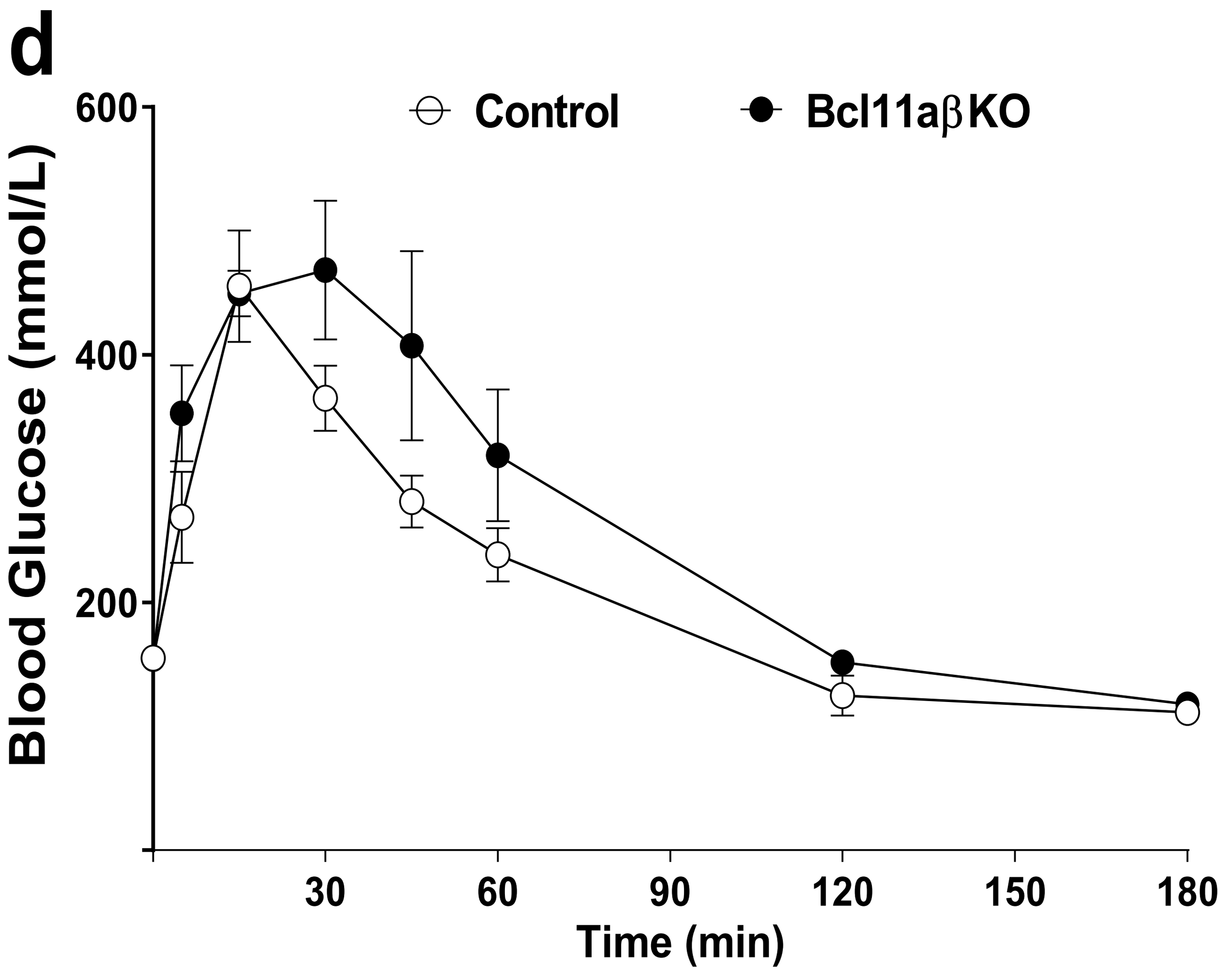

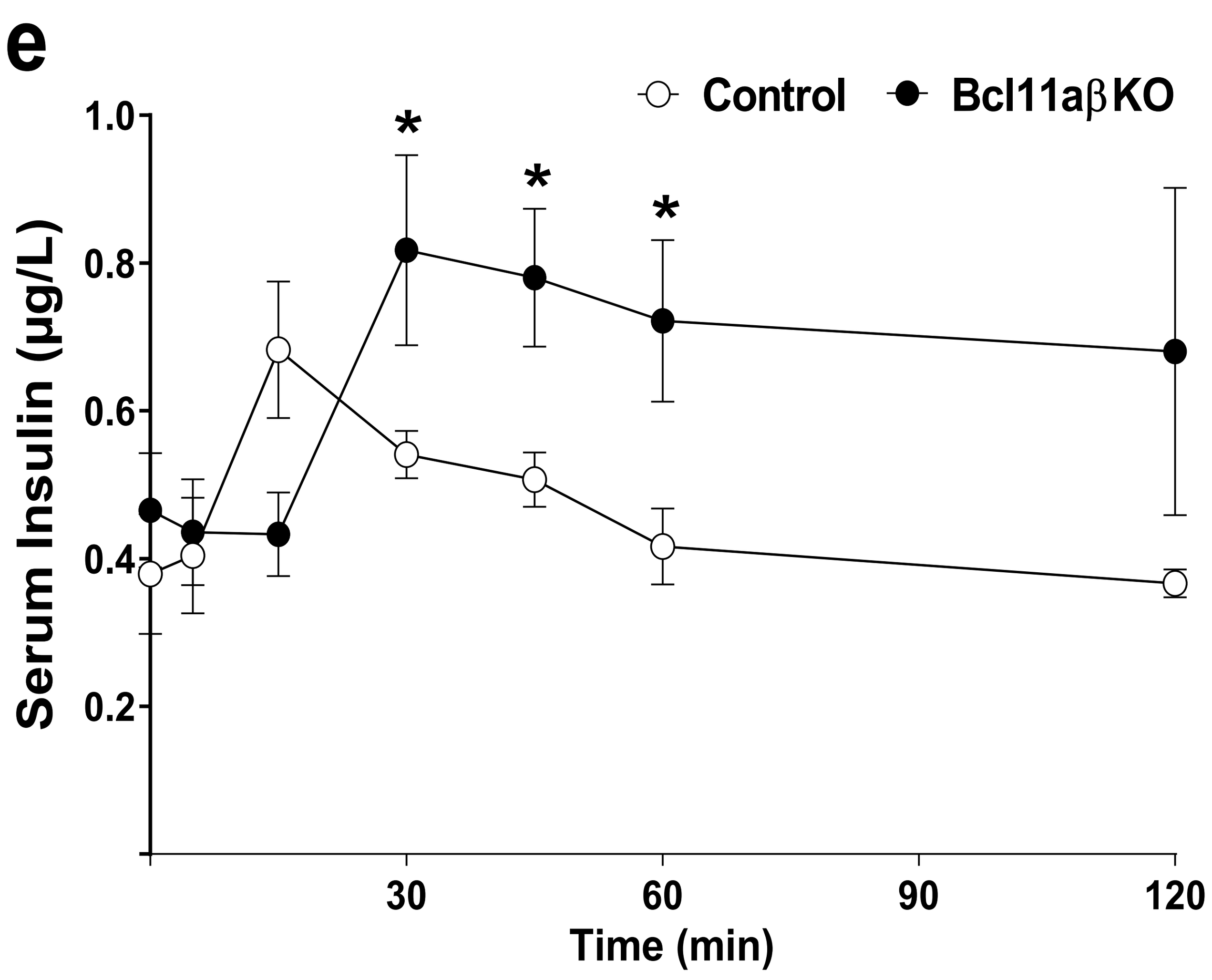

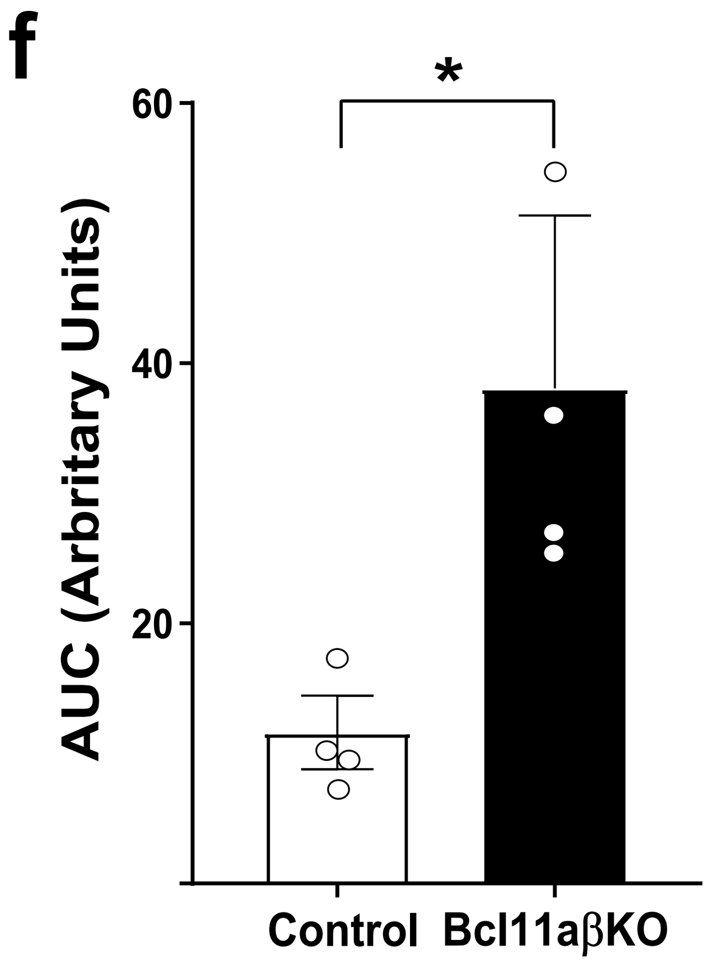

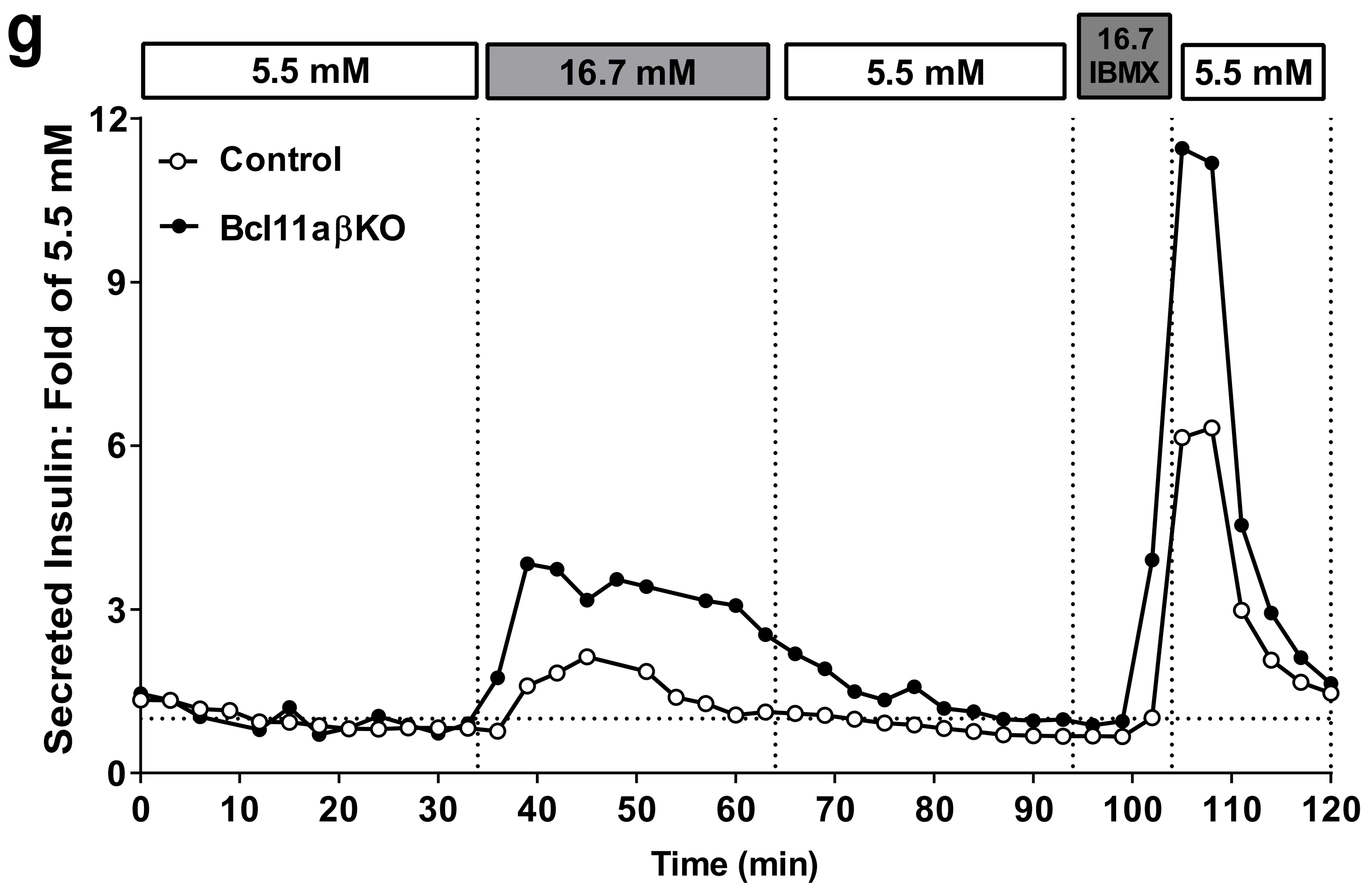

Supplementary Figure 5: (a) Donor information of the islets used to generate data presented in Figure 5.
(b) Immunohistochemical staining of mouse pancreatic sections with BCL11A (white) and Insulin (red), scale bar = 100 µm.
(c) Quatification of Bcl11a deletion in mouse islets (n=2 mice per group).
(d) Glucose tolerance test of eight week old male Control (white symbols) and Bcl11aβKO (black symbols) mice. 2 grams of glucose per kg body weight was injected into mice intra-peritoneally at t=0, (n=4 mice per group).
(e) In vivo glucose-stimulated insulin secretion of eight week old male Control (white symbols) and Bcl11aβKO (black symbols) mice, (n=4 mice per group).
(f) Area under the curve of in vivo glucose-stimulated insulin secretion graph shown in panel e, (n=4 mice per group).
(g) Insulin secretion in mouse islet perifusion assays using islets isolated from eight week old male Control (white symbols) and Bcl11aβKO (black symbols) mice.
Data presented as mean, error bars represent the standard error, and two-tailed t-tests were used to generate p values, * p<0.05.

Supplementary Figure 6 (related to Figure 5)

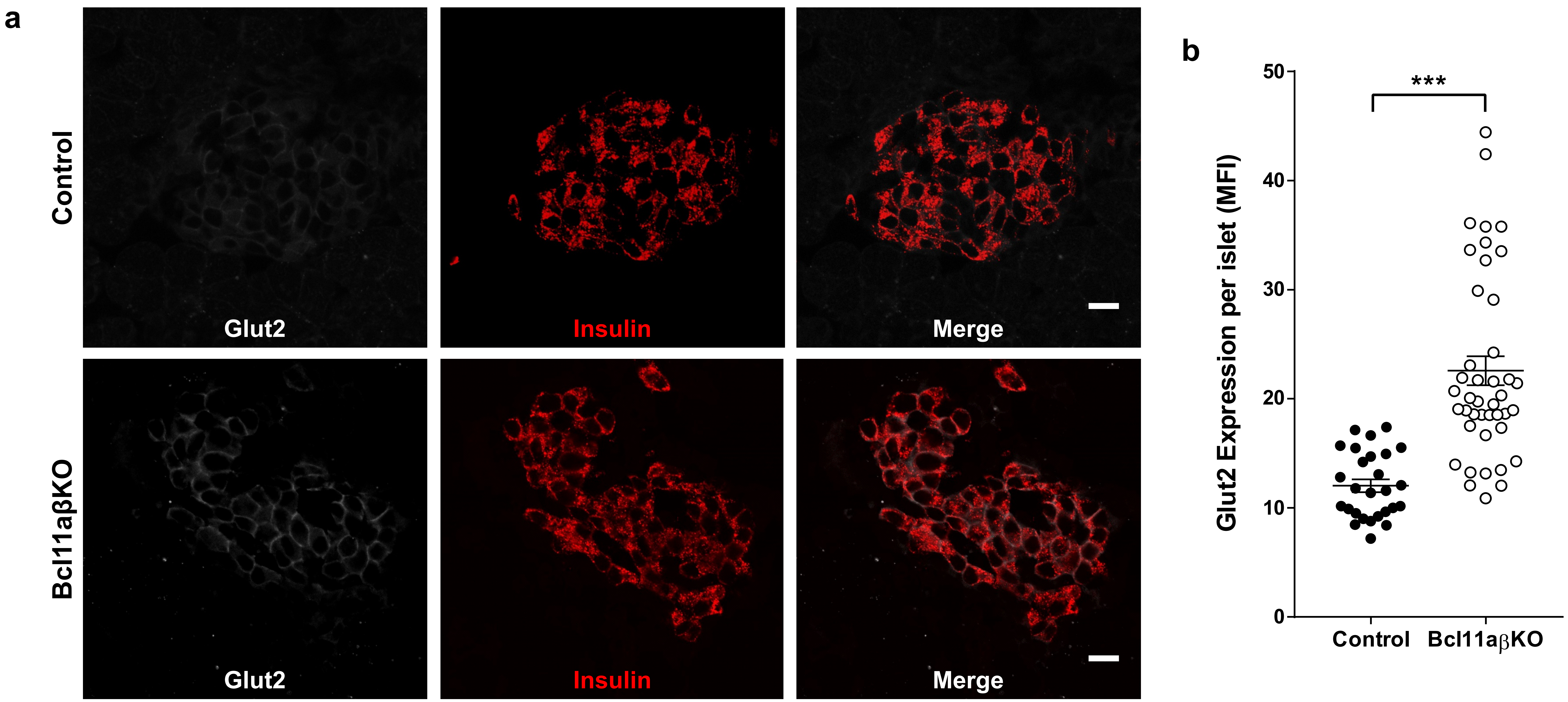

Supplementary Figure 6: (a) Immunohistochemical staining of mouse pancreatic sections with Glut2 (white) and Insulin (red), scale bar = 20 µm. **(b)** Quatification of Glut expression by Mean Fluorescence Intensity (MFI) in mouse islets (n=2 mice per group, 70 islets quatified in total). Data presented as mean, error bars represent the standard error, and two-tailed t-tests were used to generate p values, *** p<0.001.

Table 1: List of genes significantly upregulated post-BCL11A knockdown in human beta cells.

Gene	Fold Change (log2)	P value	Gene	Fold Change (log2)	P value	Gene	Fold Change (log2)	P value	Gene	Fold Change (log2)	P value	Gene	Fold Change (log2)	P value
ANKRD31	1.3527228	5.05E-06	TXNRD3	0.6735558	0.02458	ARHGEF28	0.6177510	0.028865	SLC38A2	0.5699574	0.042001	ZNF592	0.496738	0.047468
HEATR3	1.2415045	3.79E-05	ATP8A1	0.6735320	0.016495	REN	0.6173015	0.012208	WDR11	0.5698276	0.03133	ADCK1	0.4946963	0.049841
ZNF324B	1.2148419	6.34E-05	CIDEB	0.6702283	0.023438	CEP19	0.6171886	0.03998	ZZEF1	0.5696998	0.003283	DDR2	0.494645	0.042583
LRIG2	1.1713783	0.000109	NAGS	0.6699536	0.024698	PTPDC1	0.6161474	0.035647	TBC1D4	0.5695253	0.047223	RRAGA	0.493730	0.035116
SIMC1	1.1508097	6.44E-05	NUPL2	0.6680526	0.018588	HCG4B	0.6131245	0.04092	ZNF837	0.5693897	0.041903	UBR5	0.491376	0.043789
NAGPA-AS1	1.0935026	6.37E-05	STEAP1	0.6602258	0.021794	MRPL35	0.6107964	0.002083	NSMCE4A	0.5647659	0.046364	STXBP3	0.486327	0.034358
ZBTB9	1.0715642	0.00028	TOPORS	0.6597557	0.028652	BAG4	0.6096006	0.025416	SPIDR	0.5637248	0.020475	DENND4A	0.483156	0.0332
LIMS3	1.0433803	0.000608	TMEM38A	0.6593702	0.029425	RFFL	0.609298	0.031421	LINC01128	0.5619695	0.033462	NAALADL2	0.482758	0.034558
SLC38A6	0.9934824	0.001094	SLC43A1	0.6573099	0.012065	ZNF671	0.6075565	0.043195	ZGRF1	0.5613600	0.022724	LYST	0.477715	0.03271
KLHL26	0.9427275	0.000676	ZCCHC9	0.656913	0.017241	RTN4RL1	0.6068029	0.0456	SSH1	0.5604367	0.042838	CCNT2	0.4767968	0.049461
FAM171A2	0.9240238	0.002336	ZNF441	0.6566787	0.030654	RAD18	0.6064245	0.031733	SLC35F3	0.5546679	0.03059	PPARGC1B	0.474981	0.026384
LOC283856	0.8882521	0.003493	LRRC40	0.6538132	0.024199	LINC01447	0.6036617	0.041387	SNTG1	0.5520254	0.024169	POM121	0.472366	0.02603
NOL10	0.8633841	0.003	UMPS	0.6528297	0.031247	RAET1E-AS1	0.6012162	0.019127	PRDM4	0.5519033	0.036161	IFNGR2	0.472252	0.044212
SPACA6P-AS	0.8608427	0.002603	C7orf43	0.6518517	0.00634	ICAM2	0.6010740	0.029702	MIRLET7DHG	0.5514003	0.015875	PLA2G4C	0.469421	0.047581
ZNF773	0.8500356	0.004765	P3H4	0.651773	0.025198	GUSBP11	0.6004758	0.038045	WDR20	0.5513926	0.046098	AKAP8	0.467952	0.036859
FKRP	0.8324026	0.004062	RNF34	0.6517452	0.026205	PIP5K1C	0.6001526	0.025038	SMAD1	0.5513853	0.015712	CCDC97	0.467802	0.031283
NEK11	0.822206	0.006648	LINC00883	0.6491988	0.032289	C22orf42	0.5995310	0.045057	BRAT1	0.5500772	0.023281	C3orf67	0.466658	0.049295
LRRC48	0.8213830	0.003985	MRPL1	0.6472057	0.028087	HAS2	0.5986117	0.045814	UNC13B	0.5497325	0.039978	IGSF5	0.465039	0.032438
ZNF674-AS1	0.8194218	0.006657	TBCD	0.6471481	0.021247	ENAM	0.5979824	0.036054	ADNP	0.5495182	0.039488	MTMR8	0.462827	0.035413
LRRN1	0.7934620	0.002164	NR4A3	0.6469155	0.030797	SCN3A	0.5969641	0.039789	PPIL4	0.5480988	0.042413	PAK1	0.458135	0.0374
MLH1	0.7933339	0.00223	ZC3H18	0.6463698	0.01943	KIAA1656	0.5968544	0.04912	STK40	0.5473888	0.012536	BCL3	0.453876	0.04388
RNASEH2A	0.7907203	0.006425	DPP3	0.646180	0.028857	CSRNP2	0.5967428	0.037609	IKBIP	0.5453430	0.017862	GSS	0.453858	0.042967
YTHDF3-AS1	0.7728149	0.004665	GTF2I	0.6449625	0.01239	CARD8	0.5958434	0.048466	YY2	0.5432109	0.041892	NPAT	0.452948	0.024212
CTGF	0.7657431	0.011872	CLCF1	0.6443025	0.01221	SLC37A2	0.5937020	0.047908	NUAK2	0.5402197	0.022006	MSMB	0.450763	0.03722
PLEKHG1	0.7615381	0.004987	KIAA1467	0.641650	0.032967	SLC1A5	0.5929934	0.044908	KRT80	0.5341987	0.040877	SF3B4	0.448522	0.021524
SSH2	0.7448145	0.001466	BRAF	0.6414259	0.035069	USO1	0.5928833	0.004988	MAGI2	0.5322199	0.029409	SRSF7	0.442161	0.039675
PRORSD1P	0.740573	0.014248	CFAP221	0.6404613	0.034796	ITSN2	0.5926940	0.021326	ZNF792	0.5263193	0.033041	HTR2B	0.434501	0.044723
B3GNT7	0.7393813	0.015123	WIPI1	0.6366956	0.030409	PCDHB16	0.592537	0.041423	RPL19P12	0.5235712	0.027424	CDK5RAP2	0.433642	0.049631
SLC2A2	0.7363028	0.009358	TTC26	0.6354670	0.031727	RNF180	0.5915272	0.048186	NBAS	0.523256	0.02438	NUP214	0.432636	0.046017
ADAT2	0.7338706	0.015722	PLA2G6	0.6349097	0.03134	FAM86FP	0.5902831	0.035752	PKD1P1	0.5223900	0.013652	EPS15	0.428561	0.025588
DUOX1	0.7251539	0.016866	NFYA	0.6311694	0.038073	GRIP1	0.5891351	0.047703	TSPAN5	0.5199608	0.048382	ASB8	0.425048	0.038108
PDCD4-AS1	0.7159372	0.01856	DHX35	0.6304806	0.034418	MOAP1	0.5878485	0.012998	CYB5R1	0.519930	0.045117	PAPSS2	0.424118	0.012838
ACRBP	0.714437	0.00995	STXBP5L	0.6283900	0.038139	PRH2	0.5847925	0.029455	GCC2	0.5177049	0.004709	DCAF13	0.422404	0.046755
NECAP2	0.7102929	0.003398	ZSCAN2	0.6233911	0.037469	NBPF9	0.5842723	0.038557	NFYB	0.5139748	0.049088	PPP1R42	0.419619	0.046151
FBN1	0.7079598	0.015643	PTGER4	0.6231005	0.01664	ELMOD3	0.5823538	0.041777	PON2	0.5126269	0.025335	C15orf57	0.417943	0.045302
ABHD15	0.7073635	0.01416	TP73	0.6229883	0.016086	FBXO11	0.5815636	0.041887	CRELD1	0.5124043	0.009373	ADRM1	0.417364	0.045886
DNAH5	0.7058552	0.00616	TMEM185B	0.6229079	0.00786	ZNF184	0.5769067	0.028947	PKD1P6	0.5109386	0.024576	TNS3	0.394668	0.038683
ERCC6	0.7058035	0.020405	N4BP3	0.621249	0.02354	CTSE	0.5767773	0.011966	PDPK1	0.5082354	0.026706	GATS	0.393332	0.047525
ZNF75A	0.6863920	0.007077	LOC100419583	0.6208102	0.021128	NCEH1	0.5749096	0.042897	EHD3	0.504132	0.044	STXBP5	0.358573	0.018899
PDCL	0.682502	0.002898	ARSK	0.6207108	0.039433	ISY1	0.5738328	0.029875	KCNIP1	0.5039555	0.031922	UCHL3	0.344553	0.032271
GLYCTK	0.6800903	0.016833	SPESP1	0.6199222	0.015155	FANCF	0.5721338	0.01144	UXS1	0.5008109	0.024954	GLRX5	0.314755	0.046898
SMPD2	0.677433	0.021896	TAF1	0.6190034	0.017456	RALGAPA2	0.571106	0.013733	ZNF451	0.4995994	0.038722	ZBTB20	0.295490	0.042939
GAPVD1	0.673567	0.015012	HMG20B	0.6179403	0.020608	DOLK	0.5703867	0.04521	SLC9A5	0.4990996	0.026722			

Table 2: List of genes significantly downregulated post-BCL11A knockdown in human beta cells.

Gene	Fold Change (log2)	P value	Gene	Fold Change (log2)	P value	Gene	Fold Change (log2)	P value	Gene	Fold Change (log2)	P value	Gene	Fold Change (log2)	P value
LOC100379224	-1.10309	3.67E-05	DGCR6	-0.50187	0.01263	MEIS3	-0.39625	0.025429	FZD2	-0.57839	0.032886	DENND1B	-0.61532	0.043196
TMEM51	-0.970737	4.07E-05	GATSL3	-0.71426	0.0129	DYX1C1	-0.655098	0.025759	PNKD	-0.43261	0.032945	PNO1	-0.52248	0.043292
SUV420H2	-0.941085	0.000272	LOC151174	-0.60698	0.013629	TSR2	-0.49242	0.025864	RINL	-0.35288	0.032976	REL	-0.58557	0.043987
MSL1	-0.691743	0.000501	STK17A	-0.67427	0.013985	TMEM234	-0.60543	0.026091	NCOR1P1	-0.55594	0.033095	SMARCA4	-0.42405	0.044063
TGFB3	-0.970066	0.000658	LRP12	-0.72223	0.014178	MTFR1	-0.64311	0.026703	UBALD1	-0.63228	0.033468	FAM65A	-0.56462	0.044542
TUBE1	-0.682067	0.001384	MSH2	-0.74450	0.014283	TRIB2	-0.670930	0.026715	SAC3D1	-0.64000	0.033849	SIK3	-0.48712	0.044629
SGOL2	-0.930730	0.001385	TACC2	-0.71450	0.014405	C3orf38	-0.66481	0.027457	LPCAT1	-0.53728	0.034926	IFITM1	-0.57995	0.044768
STK19	-0.727177	0.001662	UCK1	-0.69069	0.014616	DAGLA	-0.66807	0.027572	CPTP	-0.64019	0.035386	PRKCI	-0.51388	0.044842
STAG3L5P	-0.927846	0.002058	IER5	-0.69582	0.015582	PRR14L	-0.61266	0.027655	MZF1-AS1	-0.61811	0.035428	GNL3L	-0.50955	0.044902
ZC3H7B	-0.903100	0.002091	F2R	-0.68260	0.015855	PIWIL4	-0.52374	0.027702	SERPINA6	-0.55105	0.035591	TRRAP	-0.59807	0.045148
TMEM134	-0.627925	0.002233	CAMKK1	-0.64321	0.016225	CUL5	-0.52258	0.027783	INAFM1	-0.63283	0.03576	POLR2J	-0.36532	0.045233
CCDC9	-0.908686	0.002787	UBE2E1	-0.63689	0.016335	CD83	-0.64699	0.027826	MEA1	-0.34441	0.03597	EXTL3	-0.55923	0.045253
C10orf88	-0.908823	0.002799	AGAP3	-0.56897	0.016493	INPP5K	-0.66774	0.028247	RQCD1	-0.55707	0.035993	WDTC1	-0.59274	0.045594
KLHL18	-0.714661	0.002819	STK38L	-0.71367	0.016951	ANKRD23	-0.65818	0.028264	LOC642366	-0.5292	0.03639	NRTN	-0.60730	0.045813
RPS6KA1	-0.874359	0.00282	RAPGEF2	-0.68146	0.01696	ARID3B	-0.661817	0.028301	ARHGAP32	-0.44134	0.036854	NLRP3	-0.52298	0.046109
POLR3B	-0.900216	0.002855	C7orf50	-0.32903	0.018615	TMEM63B	-0.605069	0.028604	POLR2D	-0.63465	0.037065	PLSCR3	-0.53651	0.046154
FZD4	-0.906366	0.002895	LOC101928847	-0.65075	0.018864	RIPK4	-0.63905	0.028737	ZNF711	-0.58646	0.037168	ST20-AS1	-0.60608	0.046455
AKT3	-0.495137	0.003165	VHL	-0.58340	0.019496	SMG6	-0.592497	0.02928	TOPORS-AS1	-0.45147	0.037582	TRAPPC13	-0.59599	0.047066
LMBRD2	-0.895204	0.003254	ARPC4-TTLL3	-0.66618	0.01995	TMEM104	-0.61281	0.029571	C1orf27	-0.53177	0.037627	USP12	-0.50248	0.047161
CCDC127	-0.699828	0.003306	RFPL3S	-0.65338	0.020068	LOC101929524	-0.457540	0.029632	CSNK2A1	-0.56466	0.037769	LOC100505942	-0.59808	0.047349
TNKS1BP1	-0.733082	0.003933	CNNM2	-0.68348	0.020086	TOE1	-0.596619	0.029701	JOSD2	-0.44305	0.037842	FSD2	-0.50327	0.047458
NFRKB	-0.854002	0.004144	PHF12	-0.61649	0.020131	HOMER2	-0.58671	0.029759	BLOC1S2	-0.36150	0.037892	BARD1	-0.50557	0.047617
TIMELESS	-0.838176	0.004389	SEC14L1	-0.57352	0.020411	EPN1	-0.51151	0.029836	CXorf58	-0.44012	0.038724	C9orf173-AS1	-0.40978	0.047702
FKBP9	-0.670889	0.004498	ECT2	-0.43578	0.020582	ANKRD20A8P	-0.66011	0.030064	KRT81	-0.42218	0.038927	TPRN	-0.57685	0.04778
MUS81	-0.784268	0.004678	AGPAT5	-0.68264	0.020621	MAATS1	-0.584967	0.030366	PLIN1	-0.43258	0.03913	SLC35E4	-0.59908	0.047968
PNPLA4	-0.674728	0.004911	CCDC102B	-0.66519	0.020786	STPG1	-0.651715	0.030438	MRPS11	-0.480486	0.030474	ZNF702P	-0.58250	0.047989
NOTCH2NL	-0.734975	0.005302	ZNF142	-0.68830	0.020949	HSPBAP1	-0.65736	0.030454	GLB1L2	-0.633250	0.030727	FAM199X	-0.52948	0.048361
DNAJC25	-0.744961	0.005478	HN1L	-0.53007	0.021176	CACNA1B	-0.65800	0.030463	SBF2-AS1	-0.628904	0.030888	RAD50	-0.52180	0.039319
CCDC77	-0.812299	0.005519	MAN2B1	-0.67545	0.021406	ADGRA1	-0.61994	0.030467	SPTSSA	-0.542440	0.031144	SBF2	-0.60794	0.039731
FHOD3	-0.772400	0.006068	ENTPD7	-0.67357	0.021818	LOC103908605	-0.62612	0.023377	PDGFRB	-0.579400	0.040001	STAT5A	-0.62212	0.039789
ARSE	-0.807986	0.006818	CAMLG	-0.40411	0.022184	OLIG1	-0.68095	0.023806	ARHGEF26	-0.625021	0.04003	ACSL4	-0.55495	0.039867
BTBD11	-0.810113	0.007411	RGL2	-0.56674	0.022693	ASB4	-0.61920	0.031332	NDUFB10	-0.301449	0.040159	TADA2B	-0.36724	0.048445
ALG3	-0.657071	0.007601	CACFD1	-0.61412	0.022717	LINC00884	-0.65355	0.031337	ANKRD50	-0.618914	0.040604	KCTD21-AS1	-0.54916	0.04856
MAPKAPK5-AS1	-0.615977	0.00768	TTL	-0.704135	0.009837	PPP6C	-0.54962	0.031699	BBS7	-0.591711	0.041174	EPB41L4A-AS1	-0.38535	0.048635
NEMP1	-0.804706	0.007871	LOC81691	-0.648392	0.010078	ZDHHC2	-0.48413	0.031883	WASIR2	-0.614973	0.041713	TPP2	-0.52038	0.048776
C1orf216	-0.73257	0.00789	ZNF709	-0.687757	0.023849	RIC1	-0.65180	0.03201	SLC35B3	-0.53499	0.042258	RFX1	-0.54673	0.048974
STAMBPL1	-0.796625	0.008796	CEP63	-0.47299	0.0241	ZNF420	-0.61811	0.032127	PLAGL2	-0.605588	0.042297	FCHSD1	-0.51234	0.048979
C14orf169	-0.781403	0.008838	NPPA	-0.646846	0.024297	HCG11	-0.62848	0.032267	PYGO2	-0.575293	0.042335	SLIT3	-0.46113	0.049129
BCKDK	-0.652024	0.010363	KLHL25	-0.68228	0.024524	MNT	-0.56846	0.032598	SLC25A19	-0.560314	0.042577	TNKS2	-0.39249	0.049781
BAP1	-0.585415	0.010382	TUBG2	-0.58411	0.024875	FTO	-0.51902	0.032645	DDX3X	-0.501463	0.042655			
HIC2	-0.729964	0.011168	MAP2K1	-0.64741	0.024954	MIR4435-2HG	-0.53095	0.032712	NPRL3	-0.548558	0.042781			
MRPL57	-0.29125	0.011764	KIN	-0.53583	0.025007	CDK8	-0.589327	0.032816	JARID2	-0.563180	0.043052			
SMG9	-0.65935	0.012027	FAN1	-0.61427	0.025293	GID8	-0.60772	0.032828	PWAR5	-0.56322	0.043185			
